# Phosphorous and Silica Recovery from Rice Husk Poultry Litter Ash: A Sustainability Analysis Using a Zero-Waste Approach

**DOI:** 10.3390/ma14216297

**Published:** 2021-10-22

**Authors:** Laura Fiameni, Ario Fahimi, Claudio Marchesi, Giampiero Pasquale Sorrentino, Alessandra Zanoletti, Karen Moreira, Bruno Valentim, Georgeta Predeanu, Laura Eleonora Depero, Elza Bontempi

**Affiliations:** 1 INSTM and Chemistry for Technologies Laboratory, Department of Mechanical and Industrial Engineering, University of Brescia, Via Branze, 38, 25123 Brescia, Italy; l.fiameni001@unibs.it (L.F.); a.fahimi@unibs.it (A.F.); c.marchesi003@unibs.it (C.M.); g.sorrentino002@unibs.it (G.P.S.); alessandra.zanoletti@unibs.it (A.Z.); laura.depero@unibs.it (L.E.D.); 2 Instituto de Ciências da Terra (ICT), Faculdade de Ciências da Universidade do Porto, Rua do Campo Alegre 1021, 4169-007 Porto, Portugal; karen.moreira@fc.up.pt (K.M.); bvvalent@fc.up.pt (B.V.); 3 Research Center for Environmental Protection and Eco-Friendly Technologies, Faculty of Applied Chemistry and Materials Science, University Politehnica of Bucharest, 1, Polizu St., 011061 Bucharest, Romania; gpredeanu@gmail.com

**Keywords:** sustainability analysis, zero waste generation, resource recovery, circular economy, poultry litter ash, phosphorous

## Abstract

Phosphate rocks are a critical resource for the European Union, and alternative sources to assure the future production of a new generation of fertilizers are to be assessed. In this study, a statistical approach, combined with a sustainability evaluation for the recovery of materials from waste containing phosphorus (P), is presented. This work proposes a strategy to recover P and silica (SiO_2_) from rice husk poultry litter ash (RHPLA). The design of experiment (DoE) method was applied to maximize the P extraction using hydrochloric acid (HCl), with the aim to minimize the contamination that can occur by leachable heavy metals present in RHPLA, such as zinc (Zn). Two independent variables, the molar concentration of the acid, and the liquid-to-solid ratio (L/S) between the acid and RHPLA, were used in the experimental design to optimize the operating parameters. The statistical analysis showed that a HCl concentration of 0.34 mol/L and an L/S ratio of 50 are the best conditions to recover P with low Zn contamination. Concerning the SiO_2_, its content in RHPLA is too low to consider the proposed recovery process as advantageous. However, based on our analysis, this process should be sustainable to recover SiO_2_ when its content in the starting materials is more than 80%.

## 1. Introduction

Recycling and recovery of resources from waste and industrial by-products are priority goals, and can be achieved by applying green chemistry principles [1]. In the last year, the reduced availability of mined materials [2], due to the pandemic [3], has highlighted the urgency to improve resource recycling more than ever [4,5].

Thus, great attention is devoted to the “zero waste strategy”, which is defined, by the Zero Waste International Alliance (ZWIA), as “designing and managing products and processes systematically to eliminate the waste and materials, conserve and recover all resources and not burn or bury them” [6]. The final aim is to reduce the waste that is landfilled and promote an overall responsible management of materials. New technologies are needed to support the decision makers in developing suitable policies towards the transition to a circular economy [7,8].

The agricultural sector has a key role in the growth of the bio-economy, since it is a major producer of biomass for feed, food, and energy [9]. In particular, phosphorus (P) can be recovered as struvite from breeding and dairy processing wastes, and used as a fertilizer [10,11].

Each year, in the world, about 3 × 10^7^ tons of biomass waste is generated by rice husk [12]. Rice husk is the coating on a seed and is a by-product of the milling process that separates the rice kernel. Rice husk has been proposed as an alternative to wood shavings and sawdust as bedding material in poultry houses [13,14]. However, rice husk is finally mixed with poultry litter, generating waste that is difficult and complex to manage.

An alternative to exploiting bio-waste is its thermal conversion by incineration, reducing its volume and eliminating toxic organic substances and pathogens [15,16]. The obtained rice husk poultry litter ash (RHPLA) is a P-rich by-product that may be directly applied as a fertilizer or used to obtain other P compounds [16,17], which is usually necessary, since ashes may release harmful substances into the environment [18,19]. The use of ashes to recover P is considered a winning strategy for saving natural resources and implementing new industrial symbiosis [20], where the waste from one industry can be considered a resource for another industry [21], such as in natural ecosystems, where there are no wastes, but only resources and products [22].

RHPLA also contains silica (SiO_2_) that derives from the rice husks. SiO_2_ is a raw, valuable material used in pharmaceuticals, cosmetics, paint, semiconductors, catalysts, glass, and adsorbent products. SiO_2_ was also proposed for stabilizing the heavy metals in fly ash [23,24]. Therefore, the economic viability of RHPLA recycling may be investigated, evaluating the possibility to simultaneously recover SiO_2_ and P.

Several wet-extraction methods have been proposed to recover P from incinerated sewage sludge ash (ISSA) because of the low energy requirement [25,26], with respect to the techniques, involving thermal annealing [27]. However, attention must be paid to heavy metals (such as Pb and Zn), often present in ISSA, which may limit the use of the products as fertilizers [28]. In this case, a purification step is needed [29].

Therefore, it is mandatory to evaluate the sustainability of the overall recovery process because the use of chemicals and/or high-energy treatments can jeopardize the environmental benefits of the recovery of the material.

It was claimed that producing SiO_2_ gel from rice husk ash by alkaline solubilization is more affordable than conventional SiO_2_ gel processing, which requires high-energy smelting operations [30,31]. However, only a few papers evaluate the sustainability of the recovery strategies for different materials [32,33]. 

Optimization surely plays an important role in ensuring the effectiveness and efficiency of any process [34,35]. The response surface method (RSM) is a powerful tool to analyze independent variables and to predict the observed response, as exactly and precisely as possible, at points within the experimental domain where no experiments were performed, thus finding the best conditions under uncertainty conditions, reducing the ambiguity. In particular, the design of experiments (DoE) method has been widely used in environmental studies [36].

Design-based modelling of experiments can be an effective instrument to assess the sustainability of a chemical process, since it allows the effect of the critical parameters to be understood, such as the molar concentration of the acid, and the liquid-to-solid ratio of the solution. In this study, the goal is to provide a cost-effective and sustainable solution to tackle the problem of the co-dissolution of trace elements with P in acid soils (e.g., P maximization and heavy metal minimization at the same time) [37]. A procedure for the evaluation of the sustainability of a process is defined and applied for the first time, to obtain P and SiO_2_ gel from RHPLA (see the study by Fiameni et al. [17]). The DoE, based on the central composite design (CCD) approach that integrates, as responses, the amount of P recovered (as a nutrient to maximize) and the amount of Zn (as a typical heavy metal to be minimized), is considered.

**Figure 1:**
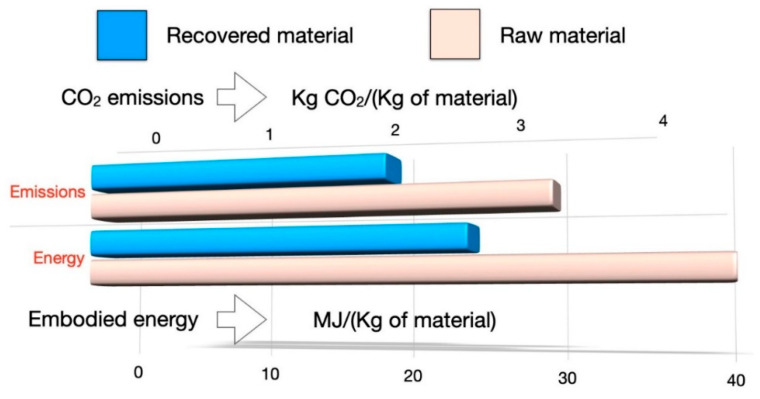
The basis of ESCAPE method, i.e., the embodied energy (EE) and the carbon footprint (CF) of recovered materials evaluated in comparison to the corresponding values for a natural resource (raw material). The method compares energies and emissions involved in the treatment of a by-product and corresponding values necessary for the synthesis of the raw material, to evaluate the sustainability. The most sustainable materials have the lowest EE and CF values. In this example the recovered material shows lower CO_2_ emissions and EE in comparison to the corresponding raw material.

Moreover, the ESCAPE (evaluation of sustainability of material substitution using carbon footprint by a simplified approach) method, proposed by Bontempi [22,38], and the sustainability indicator so-called SUB-RAW index (see Section 2.6) are applied. With this approach, we compare the potential environmental impact of a material extracted from a secondary source with the corresponding material from a natural source (as exemplified in Figure 1), considering the energies and the emissions involved in the production of the material. With the aim to optimize the whole process, the SUB-RAW index has been used as a third response to be maximized. Thus, an overall study is proposed, which combines the statistical and environmental approaches, allowing the optimization of the recovery process at the laboratory scale. 

We believe that this novel approach that integrates statistical methods and the SUB-RAW index may strongly contribute to evaluating the sustainability of any recovery process. 

## 2. Materials and Methods

### 2.1. Samples Origin

Ash samples were collected at the incinerator of Campoaves (Figueira da Foz, Portugal) and their detailed characterization is reported in Fiameni et al. [17] and Fahimi et al. [39]. The RHPLA consists of both bottom and fly ashes. In this study, only fly ash was used as collected by the “Economizer System” and named ECO. This waste is mainly composed of potassium (K_2_O = 33.1%), sulphur (SO_3_ = 17.2%), phosphorus (P_2_O_5_ = 12.7%) and calcium (CaO = 12.0%), with a SiO_2_ content greater than 12.5% [17].

After sampling, the ash was oven-dried at 50 °C until a constant weight was reached, then manually sieved using a 0.5 mm mesh. The size fraction >0.5 mm was placed in a water column. The sunk particles were then collected and oven-dried at 50 °C until a constant weight was reached.

The collected fraction was used for the leaching experiments because of the relatively high SiO_2_ percentage and the low heavy metals content (i.e., As, Cd, Hg, Cr(VI), Ni, Cu, Pb, and Zn) that may compromise its use as fertilizer [40]. While Zn content is quite high, about 1200 mg/kg or more [39], other metals are in trace and do not constitute an issue in the recovery of P.

### 2.2. The Recovery Process under Study

**Figure 2:**
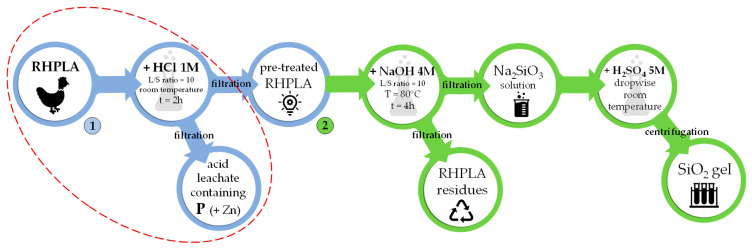
P (1) and SiO_2_ (2) recovery processes, as reported by Fiameni et al. [17].

Figure 2 shows the P and SiO_2_ recovery scheme, composed of two leaching processes. The first process (in blue in Figure 2) is the RHPLA acid leaching by HCl (1 M), and aimed to recover P. The second process (in green in Figure 2) is composed of several steps to recover the residual SiO_2_ as SiO_2_ gel by using NaOH (4 M) and H_2_SO_4_ (5 M). This process was described in detail in Fiameni et al. [17].

The tests of the first process were performed using 5 g of ECO sample stirred with HCl solution for 2 h at room temperature. Afterwards, the samples were filtered through a nylon membrane with a pore size of 0.45 µm, and the leachate solutions were analysed. The second process was not experimentally tested, while it was considered in the sustainability evaluation of the potential overall recovery (see Section 3.3) because it was still not optimized (only the feasibility studies are reported in Fiameni et al. [17]).

### 2.3. Preliminary Experimental Assessments

The recycling of macronutrients contained in RHPLA, e.g., P and potassium (K), for application as fertilizers, was already suggested [41]. However, the recovery of nutrients from RHPLA must consider the legal constraints of the heavy metals content of the recovered materials [40]. Thus, the extraction of P from the ash by a wet chemical method was investigated considering the process proposed by Fiameni et al. [17], where acid leaching technology was used as pre-treatment for the recovery of amorphous SiO_2_. 

Hydrochloric acid was chosen based on previous results [17], to propose a first attempt regarding the investigation of P recovery through acid leaching of RHPLA. This method is already applied for ISSA, for which a broad combination of types and concentrations of extractant, liquid-to-solid (L/S) ratio and contact time was already investigated in literature [25]. It was shown that an equilibrium time of 2 h is usually appropriate. Thus, in the tests, it was fixed at 2 h, while the effect of the acid concentration and the L/S ratio on the recovery efficiency was evaluated.

The parameters were optimized to obtain a solution with the highest P content and the lowest Zn contamination.

### 2.4. P Quantification by Spectrophotometric Method

After the acid leaching, the P concentrations on the eluate were evaluated by the spectrophotometric method proposed in Fiameni et al. [17] and adapted from the Romanian standardized method [42]; namely, the ECO bulk solid sample was mineralized with a mixture of concentrated acids 2:1/H_2_SO_4_:HNO_3_, filtrated and diluted with MilliQ water (Millipore DirectQ-5 TM, Millipore S.A.S., 67120, Molsheim, France). The precursor ammonium molybdate [(NH_4_)_6_Mo_7_O_24_∙4H_2_O] was mixed with H_2_SO_4_ 6 N. The produced blue phosphomolybdate complex was used as the colorimetric indicator of the P concentration in the sample. This procedure of P quantification was adopted for all 17 leachates, appropriately diluted. Spectrophotometry measurements were performed by a QE65000 spectrophotometer (Ocean Optics) suitable for measuring absorbance in the visible and near-infrared regions of the spectrum, using a polystyrene cuvette (10 mm optical path). A blank solution prepared with the same quantities of reagents and a suitable volume of MilliQ water was used as a reference. The calibration curve (absorbance vs. P content in mg/L) was obtained by standard solutions of potassium dihydrogen phosphate (KH_2_PO_4_).

### 2.5. Zn Quantification by Total Reflection X-ray Fluorescence (TXRF) Analysis

ECO bulk specimen was digested, and the Zn concentration (mg/kg) was evaluated. The procedure for sample preparation was mentioned by Assi et al. [43]; namely, 0.25 g of ash sample was placed in a Teflon vessel with a mixture of 6 mL of HNO_3_ (≥65%), 2 mL of HCl (37%) and 2 mL of HF (48%), and digested (twice as replicate to confirm the numerical output data) using a CEM SPD automated microwave digestion system (CEM, Charlotte, NC, USA). The complete digestion of the ash was realized thanks to an automatic procedure, with the following temperature ramp: 10 min to reach 200 °C, then 10 min at 200 °C, and in the end 10 min to bring the temperature back to room temperature. Finally, the digested mixtures were transferred to 50 mL polyethylene flasks and diluted to the mark with MilliQ water.

A stock solution of 1 g/L Ga in nitric acid (Ga inductively coupled plasma (ICP) standard solution, Fluka, Sigma Aldrich, Darmstadt, Germany) was used as an internal standard. Approximately 0.010 g of 100 mg/L of Ga standard solution was added to the properly diluted sample solutions, obtaining a final concentration of 1 mg/L Ga. The specimens were prepared considering the linearity range of quantification as mentioned by Borgese et al. [44]. Three plexiglass reflectors were prepared for each sample specimen and irradiated for 600 s of lifetime. Elemental chemical analysis of the digested and leachate solutions was performed using a S2 Picofox system (Bruker AXS Microanalysis GmbH, Berlin, Germany) equipped with a Mo tube (operating at 50 kV and 750 mA) and a silicon drift detector (SDD). The TXRF spectra were analyzed with instrumental software, and routine deconvolution based on mono-element profiles was applied to evaluate the peak areas.

### 2.6. ESCAPE Approach for Sustainability Evaluation

ESCAPE is based on the SUB-RAW index, a simple, direct, and versatile dimensionless index, which allows the environmental impact evaluation of waste or by-product materials if used in substitution of natural resources. It was recently used in different concerning P-based waste recovery strategies [27,32]. The SUB-RAW index is based on the following two parameters [22]: (i) the embodied energy (EE) that includes the energies directly and indirectly needed for the production of 1 kg of material; (ii) the carbon footprint (CF) that represents the equivalent mass of greenhouse gases released into the atmosphere when 1 kg of material is produced (equivalent kg of CO_2_). The parameters are calculated in MJ and kgCO_2_, respectively. They can be normalized to a reference system defined arbitrarily, and in this study, the EE and CF are normalized with respect to 1 kg of P produced (process 1) or SiO_2_ produced (process 2).

The EE and CF of reagents are reported in several databases and in this work CES Selector 2019 integrated with its commercial database [45] and open LCA [46] with free database from Ecoinvent v. 3.3 [47] were used. The “Eco Audit Tool” of CES Selector 2019 combines user-defined input with EE and CF values, processing energy and transport type to create the energy breakdown [48]. This tool was used for the calculation of EE and CF associated with the total operating power consumption (expressed in Watt, W) for moving mechanical components (mixers, crushers, mills, vacuum filters, etc.) or heating (for thermal annealing). The associated EE and CF of chemicals and water dosage were also considered.

The EE and CF parameters can be evaluated for each step of the considered technology, and the SUB-RAW index was calculated by using Equation (1) [22], as follows:

SUB-RAW index = [log(EE_RAW_)/(MJ/kg) − log(EE_SUB_)/(MJ/kg) + log(CF_RAW_) − log(CF_SUB_)]/2 (1)

EE_RAW_ and CF_RAW_ are the EE (MJ/kg) and CF (kgCO_2_/kg) of the reference process, i.e., the SiO_2_ extraction from diatomite (DE) or P extraction from the phosphate rocks (PR), while EE_SUB_ and CF_SUB_ are the EE (MJ/kg) and CF (kgCO_2_/kg) of the SiO_2_ or P extraction process from the RHPLA. The logarithm in the formula allows a direct and simple comparison considering an average of the environmental emission impact and energy consumed. The basic principle of the ESCAPE method is presented in Figure 1.

The assumptions for the calculation of EE and CF values are reported in the Supplementary Materials (from Table S1 to S12).

If the SUB-RAW index has a positive result, the proposed substitute material can be considered as more sustainable than the reference material. On the contrary, a negative index means that the proposed material is more onerous than the reference material.

In this work, the SUB-RAW index is applied for the sustainability evaluation of the recovery processes (1 and 2) explained in Section 2.2.

### 2.7. Design of Experiments (DoE)

Response surface tool is a set of experimental design methodologies that allow the response of a system to be studied in a clearer and more understandable way. Thus, the influence of several variables on the response of interest is investigated and the response can be optimized [49]. A face-centered central composite design (CCD) was adopted for the experiments, in order to investigate the optimal conditions for a chosen response and the curvature of the model between the factors [50]. The experiments were organized into 17 tests, with the following two factors being investigated: the molar concentration of the leaching agent (A) and the L/S ratio (B). In detail, two replicates of factorial points and five for central points were carried out. The choice of this design was based on a balance between good timing feasibility in the laboratory and an acceptable statistical power of the model. HCl concentration was considered in the range of 0.1–1 mol/L and L/S ratio in the range of 10–50 mL/g. Details of the 17 trials are outlined in Table 1. The considered responses were P and Zn concentrations, and the SUB-RAW indeces. The goal was to maximize the amount of P and the SUB-RAW index, and to minimize the Zn content. Design Expert 12.0.0 (Stat-Ease Inc., Minneapolis, USA) was used for performing statistical analysis in accordance with principles of the analysis of variance (ANOVA).

## 3. Results and Discussion

### 3.1. Experimental Results

**Figure 3:**
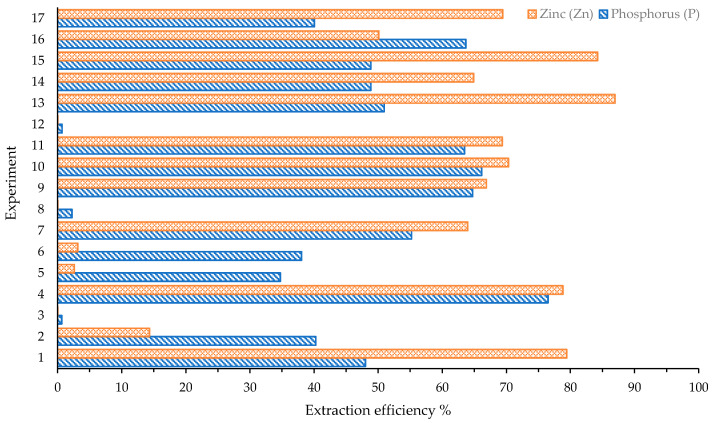
Extraction efficiency for phosphorus (P) and zinc (Zn), obtained from the results of colorimetric and TXRF analysis of the acid leachates.

Figure 3 presents the P extraction efficiency (PEE) and Zn extraction efficiency (ZEE) for the 17 experiments. The maximum PEE value, equal to 76.5%, is reached by test no. 4, but, at the same time, the ZEE is over 70%. The minimum values for ZEE (<5%) are obtained in tests no. 5 and 6, in which the PEE values are between 35% and 40%. It was reported that, for ISSA, the chemical composition and the phases of the ash can significantly affect the extraction efficiency. Thus, the same operating conditions applied to different ashes do not guarantee similar results [25]. This is the reason why we investigated the acid leaching behavior of RHPLA especially. The low value obtained for PEE suggests that polyphosphate and organophosphorus compounds can only be determined via phosphomolybdate complexation if they are converted to molybdate reactive orthophosphate, formed by sulfuric acid hydrolysis [51]. For the same reason, investigating the sulfuric lixiviation of RHPLA should be an advantageous forthcoming activity.

However, in tests no. 2, 3, 5, 6, 8, 12, and 16, PEE is greater than ZEE.

### 3.2. Statistical Analysis

The data shown in Figure 3 were submitted to ANOVA. During the preliminary statistical evaluation, test no. 2 was identified as an outlier, both for the P and Zn concentration obtained after acid leaching, probably due to the sampling. Therefore, the data of test no. 2 were not included.

#### 3.2.1. Phosphorus Extraction

**Figure 4:**
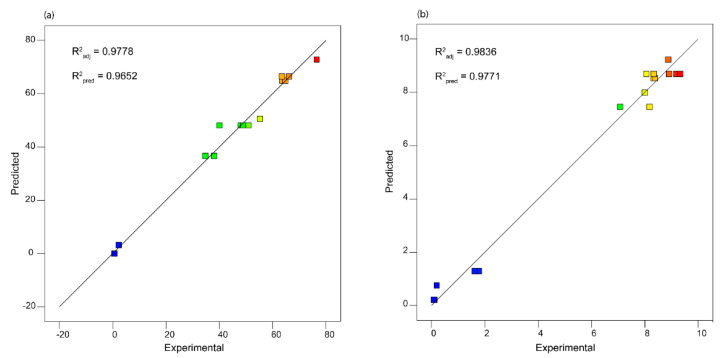
Comparison of the experimental results with predicted results by the model. P extraction (**a**) and Zn extraction (**b**). The points are colored on a scale according to the considered response starting from blue (minimum value) to red (maximum value).

To assess the capability and feasibility of the model, the correlation coefficient (R^2^) and the analysis of variance were considered. The correlation coefficient was 0.985, while the adjusted R^2^ and predicted R^2^ values were 0.98 and 0.97, respectively; therefore, the model can be reliable and stable. Figure 4a represents the predicted values using the model versus the experimental values of PEE. The model equations are reported in Supplementary Materials, Equations (1) and (2).

The ANOVA for the regression model is presented in Table 2. The Fisher test value (F-value) for the model was found to be 133.13; this means that the model was highly significant, with a *p*-value less than 0.0001. In this case, A, B, AB, A^2^, and B^2^ are significant model terms. Finally, the lack of fit F-value of 2.32 implies that the lack of fit is not significantly relative to the pure error. There is a 16.06% chance that such a large lack of fit F-value could occur due to the noise (Design Expert 12.0.0, Stat-Ease Inc., Minneapolis, MN, USA).

#### 3.2.2. Zinc Extraction

The second goal of the process was to minimize the Zn extraction. The correlation coefficient (R^2^) and ANOVA were used to test the feasibility and the reliability of the model. The R^2^ calculated in fitting was 0.99, while the adjusted and predicted R^2^ were 0.98. Thus, the model can be considered reliable and stable. The model equations are reported in Supplementary Materials, Equations (3) and (4). In Figure 4b, the predicted values using the model versus the experimental values are shown. The ANOVA is presented in Table 3. Since the model F-value is 300.07, the model is significant; this is confirmed by the *p*-value of less than 0.0001. In this case, A, B, and A^2^ are significant model terms, as indicated by the *p*-value. As for the P extraction, the lack of fit is not significantly relative to the pure error, as confirmed by the F-value of 1.03 and the p-value of greater than 0.05. The response surfaces for P and Zn extraction are shown in Supplementary Materials, Figures S1 and S2.

#### 3.2.3. SUB-RAW Index

Considering the SUB-RAW index as a response, the ANOVA results are presented in Table 4. The model is significant (F-value = 117.12), but the lack of fit is also significant (F-value = 7.77). This means that the model cannot optimize the response. Thus, the model fails to adequately describe the functional relationship between the experimental factors and the response. This inconsistency is probably due to the procedure of the SUB-RAW index calculation that was considered as an experimental result, such as PEE and ZEE. However, this index results from a numerical computation. Therefore, the model cannot be constructed in a reliable way because a previous optimization for the SUB-RAW index is necessary. These whole considerations could be a good starting point for forthcoming studies, not only for the development of an optimized SUB-RAW index for this process, but also for an improved standard method of calculation.

#### 3.2.4. Response Optimization

Since the SUB-RAW index cannot be optimized, optimization was only considered for PEE and ZEE. The model was validated considering these two factors, with the aim of maximizing the P extraction and, simultaneously, minimizing the Zn content. The optimization had to consider that the factors A and B are directly correlated with both responses. A good compromise was found in the following conditions: HCl concentration of 0.34 mol/L and L/S ratio of 50. In these conditions, we can reach 61.3% for PEE and 44.2% for ZEE.

### 3.3. Sustainability Analysis

Since the statistical evaluation of the SUB-RAW index was not reliable, we considered a comparison between the most sustainable test and the most efficient test. The sustainability analysis of the 17 tests (in terms of EE and CF) was performed and compared to the similar process reported by Habashi et al. [52], in which the P is recovered from PR. According to Equation (1), the 17 SUB-RAW indeces were calculated. In Supplementary Materials (Table S13), all the values are reported, and the best SUB-RAW index is obtained by test no. 9, with a value equal to −0.34.

Table 5 shows the results of EE and CF, concerning the P recovery from PR as raw material and from RHPLA of tests no. 4 and 9. Test no. 4 showed the highest PEE (76.5%), despite a less sustainable SUB-RAW index (−0.70) than that obtained in test no. 9. The reference process [52] showed a % of P recovery of about 99% (out of the 13.62% available in the PR), thanks to the involvement of beneficiation agents that were not considered in the SUB-RAW calculation, because no data regarding their concentration were reported by the reference. However, despite the negative SUB-RAW index, in terms of the EE and CF referred to in the starting material (1 kg of RHPLA), test no. 9 requires lower EE (28.2 MJ/kgRHPLA) and CF (1 kgCO_2_/kgRHPLA), in comparison to the reference process (EE_raw_ = 60.99 MJ/kgPR and CF_raw_ = 2.08 kgCO_2_/kgPR)_._ Since the approach requires a final normalization step from 1 kg of RHPLA to 1 kg of recovered material (P), the lower P grade contained in RHPLA (4.56%) leads to a negative SUB-RAW index. This problem would be overcome if this analysis had considered other types of agricultural animal-derived ashes, such as meat and bone ash, which has a P content of about 18% [53].

**Figure 5:**
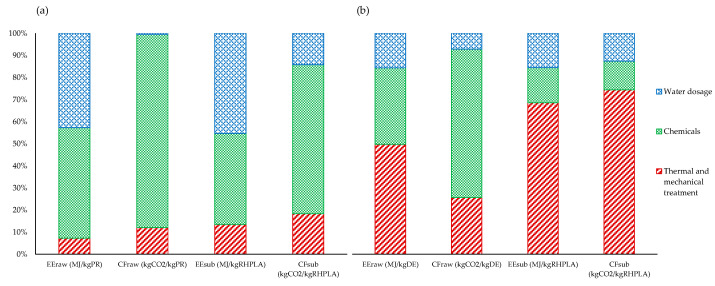
Bar chart results of the embodied energy (EE) and carbon footprint (CF) for the raw process (raw) and the new proposed process (sub), showing the relative contribution of the single procedure steps (water dosage, chemical reagents, thermal and mechanical treatment). (**a**) Represents the case of test no. 9 in process 1 (devoted to P recovery), while (**b**) represents process 2 (devoted to silica recovery).

In Figure 5a, PR and test no. 9 are compared. It results that the chemicals and water quantities represent the greatest contributions to EE, whereas, for CF, the contribution of chemicals is more relevant compared to the contribution of the thermal and mechanical treatments, and the quantity of water used.

In the DoE study, the SUB-RAW index was calculated for the 17 tests and considered in the optimization, together with the PEE and ZEE percentages. The input parameters were the HCl molar concentration and the L/S ratio.

The values of the EE and CF of the second process, to recover SiO_2_, described in Section 2.2, are 128 MJ/kgRHPLA and 6 kgCO_2_/kgRHPLA, respectively. The identified reference process considers the extraction of SiO_2_ from the DE [54], since it has been suggested to be one of the most promising, stable, and steady sources of SiO_2_. The calculated values of EE_raw_ and CF_raw_ for the reference process are 99 MJ/kgDE and 10.37 kgCO_2_/kgDE, respectively.

As reported in Table 6, the EE and CF values calculated for the reference process and the process proposed for recovering SiO_2_ are comparable. More than 50% of the EE of both processes is due to the mechanical and thermal treatments, and the value of EE_sub_ is greater by about 38 MJ/kg, in comparison to the value corresponding to EE_raw_. The value of EE_sub_ could be decreased by about 17 MJ by using an electric oven instead of a halogen furnace. Furthermore, the data presented in the reference process [54] did not mention the necessary step of drying the material before the alkaline treatment, weighing it properly, and defining the correct L/S ratio. Considering these facts, in terms of sustainability, the two processes are comparable.

If 1 kg of extracted SiO_2_ was considered, since, in RHPLA, the extraction efficiency is about 20% [17] (out of the 13% available in RHPLA), and in the DE, the extraction efficiency is about 31% [54] (out of the 80% available in DE), the SUB-RAW index result would be negative (−0.9), thus the results suggest that the recovery process is less sustainable. If the waste contained 80% of SiO_2_, the SUB-RAW index would be close to zero.

In Figure 5b, it is shown that, for the extraction process of SiO_2_ from RHPLA, the highest contribution to both EE_sub_ and CF_sub_ is due to the thermal and mechanical treatments. Instead, in the reference process, only EE is determined by thermal and mechanical treatments, while the CF value is mainly related to the use of chemicals.

## 4. Conclusions

This paper has reported a novel integrated approach that aimed to evaluate the potential correlation between the input parameters (HCl molar concentration and L/S ratio) and the responses (SUB-RAW index, PEE, and ZEE), in the frame of P and SiO_2_ recovery from RHPLA by wet extraction. This is the first study to investigate the possibility of recovering valuable elements (P) by RHPLA, minimizing the metal contamination.

The good stability of the models for PEE and ZEE allowed the optimization of the process to be appraised, in terms of the maximization of P recovery and minimization of the main heavy element (Zn). The operating conditions suggested by the experimental design are 0.34 mol/L HCl concentration and 50 L/S ratio, to allow PEE equal to 61.3% and ZEE equal to 44.2%. On the other hand, sustainability analysis has shown that the most sustainable operating conditions can be achieved by adopting 1 mol/L of HCl and an L/S ratio equal to 10, and the analysis needs to start from a by-product material that has a minimum P content of 10%. In this case, the PEE would be about 65%, without considering the minimization of the heavy metal content.

The results show that the global process for amorphous SiO_2_ recovery from RHPLA is less sustainable compared to the corresponding SiO_2_ extraction from raw materials, because the SiO_2_ content in RHPLA is too low. Environmental sustainability of the proposed recovery process could be achieved in the case in which the by-product material reaches a SiO_2_ content of about 80%.

In conclusion, from an environmental point of view, a good practice to manage RHPLA can consist of P recovery by wet chemical extraction, to eventually address the solid residual as a building material (for example, for cement production), due to its content of SiO_2_.

Future studies are mandatory for optimization of the process. It is important to highlight that the RHPLA has the advantage of being a by-product that is more environmentally sustainable than raw material, which must be mined, physically treated, and milled.

## Figures and Tables

**Table 1:** Experimental design for P and Zn extraction with hydrochloric acid.

Run Order	HCl Concentration [mol/L]	L/S Ratio [mL/g]
1	0.55	30
2	0.55	10
3	0.1	10
4	0.55	50
5	0.1	50
6	0.1	50
7	1	30
8	0.1	30
9	1	10
10	1	50
11	1	50
12	0.1	10
13	0.55	30
14	0.55	30
15	0.55	30
16	1	10
17	0.55	30

**Table 2:** ANOVA analysis for response surface second-order model. The considered responses are the efficiency of P extraction.

Source	DF	SS	MS	F-Value	*p*-Value
Model	5	8488.23	1697.65	133.13	<0.0001 ^a^
A	1	5610.70	5610.70	439.98	<0.0001
B	1	779.21	779.21	61.10	<0.0001
AB	1	616.73	616.73	48.36	<0.0001
A^2^	1	1004.99	1004.99	78.81	<0.0001
B^2^	1	538.90	538.90	42.26	<0.0001
Residual	10	127.52	12.75	-	-
Lack of Fit	2	46.80	23.40	2.32	0.1606 ^b^
Pure Error	8	80.72	10.09	-	-

A = HCl concentration, B = L/S ratio, DF = degrees of freedom, SS = sum of squares, MS = mean square. ^a^ Significant at the 5% probability level, ^b^ not significant at the 5% probability level.

**Table 3:** ANOVA analysis for response surface second-order model. The considered responses are the efficiency of Zn extraction.

Source	DF	SS	MS	F-Value	*p*-Value
Model	3	206.32	68.77	300.07	<0.0001 ^a^
A	1	130.98	130.98	571.50	<0.0001
B	1	2.56	2.56	11.17	0.0059
A^2^	1	69.03	69.03	301.20	<0.0001
Residual	12	2.75	0.23		
Lack of Fit	4	0.93	0.23	1.03	0.4488 ^b^
Pure Error	8	1.82	0.23		

A = HCl concentration, B = L/S ratio, DF = degrees of freedom, SS = sum of squares, MS = mean square. ^a^ Significant at the 5% probability level, ^b^ not significant at the 5% probability level.

**Table 4:** ANOVA analysis for SUB-RAW index responses obtained from the reprocessing of the experimental data.

Source	DF	SS	MS	F-Value	*p*-Value
Model	5	6.57	1.31	117.12	<0.0001
A	1	2.26	2.26	201.39	<0.0001
B	1	0.58	0.58	51.67	<0.0001
AB	1	3.35	3.35	298.65	<0.0001
A^2^	1	0.33	0.33	29.36	0.0003
B^2^	1	0.55	0.54	48.78	<0.0001
Residual	10	0.11	0.01		
Lack of Fit	2	0.07	0.04	7.77	0.0133
Pure Error	8	0.04	0.005		

**Table 5:** Data results of the embodied energy (EE) and carbon footprint (CF) for the raw process (raw) and the new proposed process (sub) related to process 1 in the frame of ESCAPE study; it shows the comparison between the most efficient trial in terms of % P extraction (test no. 4) and the most sustainable trial (test no. 9) relative to the other trials.

Considered Processes and Materials	EE_raw_ (MJ/kgPR)	CF_raw_ (kgCO_2_/kgPR)	EE_sub_ (MJ/kgRHPLA)		CF_sub_ (kgCO_2_/kgRHPLA)	
			Test No. 4	Test No. 9	Test No. 4	Test No. 9
Thermal and mechanical process	4.43	0.25	3.80	3.80	0.19	0.19
Chemical reagents	30.51	1.82	31.94	11.62	1.90	0.69
Water dosage	26.06	0.01	39.37	12.75	0.75	0.15
TOTAL	60.99	2.08	75.11	28.17	2.84	1.02

**Table 6:** Data results of the embodied energy (EE) and carbon footprint (CF) for the raw process (raw) and the new proposed process (sub) for silica recovery.

Considered Processes and Materials	EE_raw_ (MJ/kgDE)	CF_raw_ (kgCO_2_/kgDE)	EE_sub_ (MJ/kgRHPLA)	CF_sub_ (kgCO_2_/kgRHPLA)
Thermal and mechanical treatment	49	3	87	4
Chemicals	34	7	20	1
Water dosage	16	1	20	1
Total	99	10	128	6

## Data Availability

Data is contained within the article.

## Supplementary Materials

The following are available online at https://www.mdpi.com/article/10.3390/ma14216297/s1, Figure S1: Response surface plot of % of P extraction to evaluate the effects of HCl concentration and the L/S ratio, Figure S2: Response surface plot of % of Zn extraction to evaluate the effects of HCl concentration and the L/S ratio, Table S1: Characteristic parameters of the laboratory tools for the silica extraction, Table S2: EE and CF for each reference taken in consideration for the silica gel extraction from the sub-raw material, Table S3: Main data for the step 1 for the silica gel extraction from the sub-raw material, Table S4: Main data for the step 2 (alkaline leaching) for the silica gel extraction from the sub-raw material, Table S5: Main data for the step 2 (acid dropwise) for the silica gel extraction from the sub-raw material, Table S6: Main data for the step 1 for the silica extraction from the raw material, Table S7: Main data for the step 2 (alkaline leaching) for the silica extraction from the raw material, Table S8: Main data for the step 2 (acid dropwise) for the silica extraction from the raw material, Table S9: Characteristic parameters of the laboratory tools for the phosphorus extraction, Table S10: EE and CF for each reference taken in consideration for the phosphorus extraction from the sub-raw material, Table S11: Main data for the step 1 (test n. 4) for the phosphorus extraction from the sub-raw material, Table S12: Main data for the step 1 for the phosphorus extraction from the raw material, Table S13: Values of the 17 SUB-RAW indeces calculated for the phosphorus extraction from the sub-raw material.
