# Supplementary material for: Phosphorous and Silica Recovery from Rice Husk Poultry Litter Ash: A Sustainability Analysis Using a Zero-Waste Approach
Source: Materials (Basel). 2021 Oct 22;14(21):6297. doi: 10.3390/ma14216297 (PMC8585221; doi:10.3390/ma14216297)
Supplement: Supplementary file 1 [file materials-14-06297-s001.zip › materials-1390332-supplementary.pdf]

materials

Supplementary Material

Phosphorous and Silica Recovery from Rice Husk Poultry Litter Ash: A Sustainability Analysis Using a Zero-Waste Approach

1. Statistical analysis

The equation used for the P extraction as response variable was a second-order polynomial with also the interaction term Equation (1):

$$Y_1 = b_0 + b_1X_1 + b_2X_2 + b_{12}X_1X_2 + b_{11}X_1^2 + b_{22}X_2^2 \quad (1)$$

where b_i are the coefficients for linear effects, b_{ij} are the coefficients of the interaction term, and b_{ii} are the coefficients for squared effects. Y_1 is the response variable of a percentage of phosphorus extraction, and X_i are the independent variables.

The empirical relationship between the responses and the independent variables are expressed as follows Equation (2):

$$Y_1 = 48.10 + 23.69X_1 + 9.55X_2 - 8.78X_1X_2 - 21.24X_1^2 + 15.13X_2^2 \quad (2)$$

The response surface for P extraction is presented in Figure S1.

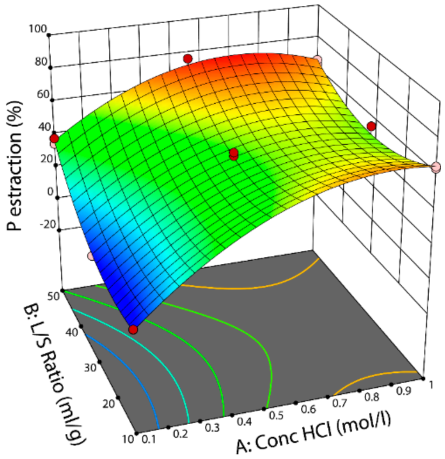

Figure S1. Response surface plot of % of P extraction to evaluate the effects of HCl concentration and the L/S ratio.

To correlate the response of Zn extraction and the independent variables a second-order polynomial equation was used. It is worth noting that, in this case, a transformation of response was required due to the non-normality distribution of the residuals with the original response; more specifically, a square root transformation was used. The second-order equation used for the prediction of the response is the following Equation (3):

$$Y_2 = b_0 + b_1X_1 + b_2X_2 + b_{11}X_1^2 \quad (3)$$

The empirical relationship between the responses and the independent variables are shown in Equation (4):

$$Y_1 = 8.69 + 3.62X_1 + 0.5383X_2 - 4.32X_1^2 \quad (4)$$

The response surface for Zn extraction is presented in Figure S2.

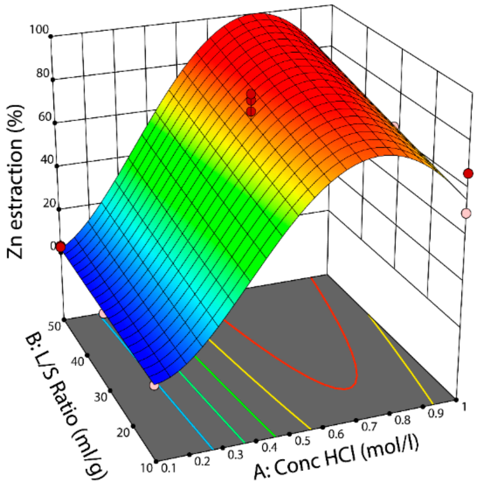

Figure S2. Response surface plot of % of Zn extraction to evaluate the effects of HCl concentration and the L/S ratio.

2. Hypothesis calculation for the SUB-RAW index

2.1. The hypothesis regarding starting materials

1. All the data regarding the Embodied Energy (EE) and Carbon Footprint (CF) of the reagents, water (ultrapure and not) and raw materials are taken from the database "Environmental Footprint" that is a subset of ECOINVENT 2019 used in the software openLCA 1.10.3.;
2. Considering that the RHPLA is an incineration treatment by-product, so it is waste, its initial EE and CF are considered null while for the Phosphate Rock (PR) and the Diatomite (DE) these values are not null considering the mining and drying process before the laboratory treatment.

2.2. The hypothesis regarding laboratory equipment

1. The filtration is done under slight suction through Whatman filter paper grade 589/2 (pore size 4–12 mm) or nylon membrane pore size 0.45 μm. The flow filtration is calculated considering the time to fill up a container of 1l simulating the filtration process, and it results equal to 18.45s. Then this flow rate value is multiplied for the time filtration of the sample used. It has been considered a time filtration value equal to 7.95 min. obtained by an average of the 17 experiments for the DOE. the tap water flow used is normalized with respect to the initial RHPLA mass in order to have the liter used for each kg of initial material used. This value so calculated is considered for all the other cases where the filtration step is used, because of the lack of information about this last value;
2. The volume of the H_2SO_4 solution used in the process C of the sub-raw material is equal to 13ml. it is assumed to use this value also for the raw material considering the similarity of the process and the quantities of the raw material and sub-raw material used for the alkaline leaching;
3. The stirring time for the dropwise step with the H_2SO_4 solution in the raw material process is assumed to equal to the one used for the sub-raw material, that is 20 minutes;
4. For all the washing steps, it is considered a value of 500 ml of ultrapure water for each 30g of material;
5. In the absence of the power curves of the instruments used for all the drying steps, the power used is calculated as a proportion considering the follow equation: $P : T = Pn : Tmax$

6. Where:
7. P is the power calculated;
8. T is the temperature used for the drying;
9. P_n is the nominal power of the electric oven;
10. T_{max} is the maximum temperature that the electric oven can reach.
11. In the raw material process, for the centrifugation step after the alkaline leaching, it is considered the follow data: NEYA centrifuge model 16-R at 2500 rpm for 15 min at 25 °C;
12. For the EE and CF of the stirring step in a heated environment the EErif and a CFrif are taken as an average between the reference value for the transition from electrical to mechanical and the one from electrical to thermal;

2.3. The hypothesis regarding the SUB-RAW index

1. In the calculation of the SUB-RAW between the RHPLA and the diatomite, it must be considered that the purification grade of the silica gel obtained from the sub-raw process is around the 80% while the one from the raw material is around 99.9% (by considering just the first part of the process);

3. Silica gel extraction

3.1. Sub-raw material process

Table S1. Characteristic parameters of the laboratory tools for the silica extraction.

$P_{stirring}$	$P_{stirring/heating}$	$P_{Centrifugation}$	P_{drying}	$P_{drying\_halogen}$	Filtration time
(W)	(W)	(W)	(W)	(W)	(min)

Table S2. EE and CF for each reference taken in consideration for the silica gel extraction from the sub-raw material.

EE_{MilliQ}	CF_{MilliQ}	$EE_{TapWater}$	$CF_{TapWater}$	$EErif_{EtoM}$	$CFrif_{EtoM}$	$EErif_{EtoT}$	$CFrif_{EtoT}$
(MJ/kg)	(kg CO_{2eq}/kg)	(MJ/kg)	(kg CO_{2eq}/kg)	(MJ/kg *W*min)	(kg CO_{2eq} /kg *W*min)	(MJ/kg *W*min)	(kg CO_{2eq} /kg *W*min)

3.1.1. Procedure for the step 1

Table S3. Main data for the step 1 for the silica gel extraction from the sub-raw material.

S/L ratio	$[HCl]_{fin}$	RHPLA mass	Density (HCl 1M)	$EErif_{HCl\,37\%}$	$CFrif_{HCl\,37\%}$
(kg RHPLA/L)	(mol/L)	(g)	(kg/L)	(MJ/kg)	(kgCO_{2eq}/kg)

The first step is to find the HCl mass present inside the solution, Equation (5):

$$m_{HCl_{in}} = m_{HCl_{fin}} = m_{HCl} = \frac{[HCl]_{fin} * PM_{HCl}}{\mathrm{S/L}} = \frac{kg}{kg\ RHPLA} \tag{5}$$

To produce the 1M HCl solution, a certain amount of 37% HCl (it is the only one present into the database mentioned at the 1st hypothesis) and MilliQ (ultrapure water) for dilution are necessary, Equations (6–7):

$$m_{sol\ HCl\ 37\%} = \frac{m_{HCl}}{0.37} = \frac{kg}{kg\ RHPLA} \tag{6}$$

$$m_{MilliQ\ to\ add} = m_{sol\ HCl\ 1M} - m_{sol\ HCl\ 37\%} = \frac{kg}{kg\ RHPLA} \tag{7}$$

the EE and CF of both the 37% HCl solution and the MilliQ used are calculated as follows, Equations (8–11):

$$EE_{HCl\ 37\%} = EErif_{HCl\ 37\%} * m_{sol\ HCl\ 37\%} = \frac{MJ}{kg\ RHPLA} \tag{8}$$

$$CF_{HCl\,37\%} = CFrif_{HCl\,37\%} * m_{sol\,HCl\,37\%} = \frac{kg\,CO_{2eq}}{kg\,RHPLA} \tag{9}$$

$$EE_{MilliQ\,to\,add} = EE_{MilliQ} * m_{MilliQ\,to\,add} = \frac{MJ}{kg\,RHPLA} \tag{10}$$

$$CF_{MilliQ\,to\,add} = CF_{MilliQ} * m_{MilliQ\,to\,add} = \frac{kg\,CO_{2eq}}{kg\,RHPLA} \tag{11}$$

Then it is possible to move on to the calculation of the EE and CF for the treatments performed in the laboratory, that are:

Stirring for t=2h at environmental temperature, Equations (12–13):

$$EE_{Stirring} = P_{Stirring} * t_{Stirring} * EErif_{EtoM} = \frac{MJ}{kg\,RHPLA} \tag{12}$$

$$CF_{Stirring} = P_{Stirring} * t_{Stirring} * CFrif_{EtoM} = \frac{kg\,CO_{2eq}}{kg\,RHPLA} \tag{13}$$

Filtration (follow the hypothesis 3), Equations (14–15):

$$EE_{Filtration} = EE_{TapWater} * m_{TapWater} = \frac{MJ}{kg\,RHPLA} \tag{14}$$

$$CF_{Filtration} = CF_{TapWater} * m_{TapWater} = \frac{kg\,CO_{2eq}}{kg\,RHPLA} \tag{15}$$

Where the $m_{TapWater}$ is the mass of tap water used by the filtration normalized on the RHPLA mass.

Washing (follow the hypothesis 6), Equations (16–17):

$$EE_{MilliQ\_Washing} = EE_{MilliQ} * m_{MilliQ_{Washing}} = \frac{MJ}{kg\,RHPLA} \tag{16}$$

$$CF_{MilliQ\_Washing} = CF_{MilliQ} * m_{MilliQ_{Washing}} = \frac{kg\,CO_{2eq}}{kg\,RHPLA} \tag{17}$$

Where the $m_{MilliQ_{Washing}}$ is the mass of MilliQ used (500 mL) by the washing, normalized with respect to the mass of starting material.

The $EErif_{EtoM}$ and the $EErif_{EtoT}$ are respectively the transformation factor of electric energy into mechanics and of electric energy in thermal.

3.1.2. Procedure for the step 2 (alkaline leaching)

Table S4. Main data for the step 2 (alkaline leaching) for the silica gel extraction from the sub-raw material.

S/L ratio	$[NaOH]_{fin}$	Density (NaOH 4M)	$EErif_{NaOH}$	$CFrif_{NaOH}$
(kgRHPLA/L)	(mol/L)	(kg/L)	(MJ/kg)	($kgCO_{2eq}$/kg)

As it was done for the step 1, the first step is to find the NaOH mass present inside the solution, Equation (18):

$$m_{NaOH_{in}} = m_{NaOH_{fin}} = m_{NaOH} = \frac{[NaOH]_{fin} * PM_{NaOH}}{\mathrm{S/L}} = \frac{kg}{kg\,RHPLA} \tag{18}$$

To produce the 4M NaOH solution, a certain amount of MilliQ is necessary, Equation (19):

$$m_{MilliQ\,to\,add} = m_{NaOH\,4M} - m_{NaOH} = \frac{kg}{kg\,RHPLA} \tag{19}$$

the EE and CF of both the NaOH solid and the MilliQ used are calculated as follows Equations (20–23):

$$EE_{NaOH} = EErif_{NaOH} * m_{NaOH} = \frac{MJ}{kg\,RHPLA} \tag{20}$$

$$CF_{NaOH} = CFrif_{NaOH} * m_{NaOH} = \frac{kg\,CO_{2eq}}{kg\,RHPLA} \tag{21}$$

$$EE_{MilliQ\ to\ add} = EE_{MilliQ} * m_{MilliQ\ to\ add} = \frac{MJ}{kg\ RHPLA} \quad (22)$$

$$CF_{MilliQ\ to\ add} = CF_{MilliQ} * m_{MilliQ\ to\ add} = \frac{kg\ CO_{2eq}}{kg\ RHPLA} \quad (23)$$

Then it is possible to move on to the calculation of the EE and CF for the treatments performed in the laboratory, that are:

Drying_halogen, Equations (24–25):

$$EE_{Drying\_halogen} = P_{Drying\_halogen} * t_{Drying\_halogen} * EErif_{EtoT} = \frac{MJ}{kg\ RHPLA} \quad (24)$$

$$CF_{Drying\_halogen} = P_{Drying\_halogen} * t_{Drying\_halogen} * CFrif_{EtoT} = \frac{kg\ CO_{2eq}}{kg\ RHPLA} \quad (25)$$

The residue was dried with the halogen electric oven for t=9h at 120 ° C, the power value used is assumed as half of the nominal one (in according with the hypothesis 7), i.e., 700W was taken because we use almost half of the maximum temperature that the oven can reach.

Stirring and heating for t=4h at 80°C (follow the hypothesis 9), Equations (26–27):

$$EE_{Stirrin/Heating} = P_{Stirrin/Heating} * t_{Stirrin/Heating} * Average(EErif_{EtoM}; EErif_{EtoT}) = \frac{MJ}{kg\ RHPLA} \quad (26)$$

$$CF_{Stirrin/Heating} = P_{Stirrin/Heating} * t_{Stirrin/Heating} * Average(CFrif_{EtoM}; CFrif_{EtoT}) = \frac{kg\ CO_{2eq}}{kg\ RHPLA} \quad (27)$$

Filtration (follow the hypothesis 3), Equations (28–29):

$$EE_{Filtration} = EE_{TapWater} * m_{TapWater} = \frac{MJ}{kg\ RHPLA} \quad (28)$$

$$CF_{Filtration} = CF_{TapWater} * m_{TapWater} = \frac{kg\ CO_{2eq}}{kg\ RHPLA} \quad (29)$$

3.1.3. Procedure for the step 2 (acid dropwise)

Table S5. Main data for the step 2 (acid dropwise) for the silica gel extraction from the sub-raw material.

S/L ratio	$[H_2SO_4]_{fin}$	Density (H_2SO_4 5M)	$EErif_{H2SO4\ 98\%}$	$CFrif_{H2SO4\ 98\%}$
(kgRHPLA/L)	(mol/L)	(kg/L)	(MJ/kg)	($kgCO_{2eq}$/kg)

Again, the first step is to find the H_2SO_4 mass present inside the solution Equation(30):

$$m_{H_2SO_{4in}} = m_{H_2SO_{4fin}} = m_{H_2SO_4} = \frac{[H_2SO_4]_{fin} * PM_{H_2SO_4}}{S/L} = \frac{kg}{kg\ RHPLA} \quad (30)$$

To produce the 5M H_2SO_4 solution, a certain amount of 98% H_2SO_4 (it is the only one present into the database mentioned at the 1st hypothesis) and MilliQ (ultrapure water) for dilution are necessary, Equations (31–32):

$$m_{sol\ H_2SO_4\ 98\%} = \frac{m_{H_2SO_4}}{0.98} = \frac{kg}{kg\ RHPLA} \quad (31)$$

$$m_{MilliQ\ to\ add} = m_{sol\ H_2SO_4\ 5M} - m_{sol\ H_2SO_4\ 98\%} = \frac{kg}{kg\ RHPLA} \quad (32)$$

the EE and CF of both the 98% H_2SO_4 solution and the MilliQ used are calculated as follows Equations (33–36):

$$EE_{sol\ H_2SO_4\ 98\%} = EErif_{H_2SO_4\ 98\%} * m_{sol\ H_2SO_4\ 98\%} = \frac{MJ}{kg\ RHPLA} \quad (33)$$

$$CF_{sol\,H_2SO_4\,98\%} = CFrif_{H_2SO_4\,98\%} * m_{sol\,H_2SO_4\,98\%} = \frac{kg\,CO_{2eq}}{kg\,RHPLA} \quad (34)$$

$$EE_{MilliQ\,to\,add} = EE_{MilliQ} * m_{MilliQ\,to\,add} = \frac{MJ}{kg\,RHPLA} \quad (35)$$

$$CF_{MilliQ\,to\,add} = CF_{MilliQ} * m_{MilliQ\,to\,add} = \frac{kg\,CO_{2eq}}{kg\,RHPLA} \quad (36)$$

Then it is possible to move on to the calculation of the EE and CF for the treatments performed in the laboratory, that are:

Stirring for t=20 min at environmental temperature (follow the hypothesis 5), Equations (37–38):

$$EE_{Stirring} = P_{Stirring} * t_{Stirring} * EErif_{EtoM} = \frac{MJ}{kg\,RHPLA} \quad (37)$$

$$CF_{Stirring} = P_{Stirring} * t_{Stirring} * CFrif_{EtoM} = \frac{kg\,CO_{2eq}}{kg\,RHPLA} \quad (38)$$

Centrifugation for t=20 min at environmental temperature (follow the hypothesis 8), Equations (39–40):

$$EE_{Centrifugation} = P_{Centrifugation} * t_{Centrifugation} * EErif_{EtoM} = \frac{MJ}{kg\,RHPLA} \quad (39)$$

$$CF_{Centrifugation} = P_{Centrifugation} * t_{Centrifugation} * CFrif_{EtoM} = \frac{kg\,CO_{2eq}}{kg\,RHPLA} \quad (40)$$

Washing (follow the hypothesis 6), Equations (41–42):

$$EE_{MilliQ\_Washing} = EE_{MilliQ} * m_{MilliQ_{Washing}} = \frac{MJ}{kg\,RHPLA} \quad (41)$$

$$CF_{MilliQ\_Washing} = CF_{MilliQ} * m_{MilliQ_{Washing}} = \frac{kg\,CO_{2eq}}{kg\,RHPLA} \quad (42)$$

Drying_halogen for t=6h at 120°C (follow the hypothesis 7), Equations (43–44):

$$EE_{Drying\_halogen} = P_{Drying\_halogen} * t_{Drying\_halogen} * EErif_{EtoT} = \frac{MJ}{kg\,RHPLA} \quad (43)$$

$$CF_{Drying\_halogen} = P_{Drying\_halogen} * t_{Drying\_halogen} * CFrif_{EtoT} = \frac{kg\,CO_{2eq}}{kg\,RHPLA} \quad (44)$$

By adding all the terms of these mechanical / heat treatments and adding the terms of the substances we obtain $EE_{RHPLA} = \frac{MJ}{kg\,RHPLA}$ e $CF_{RHPLA} = \frac{\boldsymbol{kg}\,CO_{2eq}}{kg\,RHPLA}$ which, having known the % of SiO2 extracted from RHPLA (about 0.74g of SiO_2), will be transformed into $EE_{SiO_2\_RHPLA} = \frac{MJ}{kg\,SiO_2}$ e $CF_{SiO_2\_RHPLA} = \frac{\boldsymbol{kg}\,CO_{2eq}}{kg\,SiO_2}$.

3.2. Raw material process

3.2.1. Procedure for the step 1

Table S6. Main data for the step 1 for the silica extraction from the raw material.

S/L ratio	$[HNO_3]_{fin}$	DE mass	Density (HNO_3 2.2M)	$EErif_{HNO3\,65\%}$	$CFrif_{HNO3\,65\%}$
(kgDE/L)	(mol/L)	(g)	(kg/L)	(MJ/kg)	($kgCO_{2eq}$/kg)

The first step is to find the HNO_3 mass present inside the solution, Equation (45):

$$m_{HCl_{in}} = m_{HCl_{fin}} = m_{HCl} = \frac{[HNO_3]_{fin} * PM_{HNO_3}}{\mathrm{S/L}} = \frac{kg}{kg\,DE} \quad (45)$$

To produce the 2.2M HNO_3 solution, a certain amount of 65% HNO_3 (it is the only one present into the database mentioned at the 1st hypothesis) and MilliQ for dilution are necessary, Equations (46–47):

$$m_{sol\,HNO_3\,65\%} = \frac{m_{HNO_3}}{0.65} = \frac{kg}{kg\,DE} \quad (46)$$

$$m_{MilliQ\,to\,add} = m_{sol\,HCl\,1M} - m_{sol\,HCl\,37\%} = \frac{kg}{kg\,DE} \quad (47)$$

the EE and CF of both the 65% HNO_3 solution and the MilliQ used are calculated as follows Equations (48–51):

$$EE_{HNO_3\,65\%} = EErif_{HNO_3\,65\%} * m_{sol\,HNO_3\,65\%} = \frac{MJ}{kg\,DE} \quad (48)$$

$$CF_{HNO_3\,65\%} = CFrif_{HNO_3\,65\%} * m_{sol\,HNO_3\,65\%} = \frac{kg\,CO_{2eq}}{kg\,DE} \quad (49)$$

$$EE_{MilliQ\,to\,add} = EE_{MilliQ} * m_{MilliQ\,to\,add} = \frac{MJ}{kg\,DE} \quad (50)$$

$$CF_{MilliQ\,to\,add} = CF_{MilliQ} * m_{MilliQ\,to\,add} = \frac{kg\,CO_{2eq}}{kg\,DE} \quad (51)$$

In addition, the EE and CF of the DE must also be considered, i.e., the mining process until it is brought to the laboratory counter: $EE_{DE} = \frac{MJ}{kg\,DE}$; $CF_{DE} = \frac{\mathbf{kg}\,CO_{2eq}}{kg\,DE}$;

Then it is possible to move on to the calculation of the EE and CF for the treatments performed in the laboratory, that are:

Stirring and heating for t=3h at 60°C (follow the hypothesis 9), Equations (51–52):

$$EE_{Stirrin/Heating} = P_{Stirrin/Heating} * t_{Stirrin/Heating} * Average(EErif_{EtoM}; EErif_{EtoT}) = \frac{MJ}{kg\,DE} \quad (51)$$

$$CF_{Stirrin/Heating} = P_{Stirrin/Heating} * t_{Stirrin/Heating} * Average(CFrif_{EtoM}; CFrif_{EtoT}) = \frac{kg\,CO_{2eq}}{kg\,DE} \quad (52)$$

Filtration (follow the hypothesis 3), Equations (53–54):

$$EE_{Filtration} = EE_{TapWater} * m_{TapWater} = \frac{MJ}{kg\,DE} \quad (53)$$

$$CF_{Filtration} = CF_{TapWater} * m_{TapWater} = \frac{kg\,CO_{2eq}}{kg\,DE} \quad (54)$$

Washing (follow the hypothesis 6), Equations (55–56):

$$EE_{MilliQ\_Washing} = EE_{MilliQ} * m_{MilliQ_{Washing}} = \frac{MJ}{kg\,DE} \quad (55)$$

$$CF_{MilliQ\_Washing} = CF_{MilliQ} * m_{MilliQ_{Washing}} = \frac{kg\,CO_{2eq}}{kg\,DE} \quad (56)$$

Where the $m_{MilliQ_{Washing}}$ is the mass of MilliQ used (500 mL) by the washing, normalized with respect to the mass of starting material.

3.2.2. Procedure for the step 2 (alkaline leaching)

Table S7. Main data for the step 2 (alkaline leaching) for the silica extraction from the raw material.

**S/L ratio**	**$[NaOH]_{fin}$**	**Density (NaOH 2.5M)**	**$EErif_{NaOH}$**	**$CFrif_{NaOH}$**
[kgDE/L]	[mol/L]	[kg/L]	[MJ/kg]	[$kgCO_{2eq}$/kg]

As it was done for the step 1, the first step is to find the NaOH mass present inside the solution, Equation (57):

$$m_{NaOH_{in}} = m_{NaOH_{fin}} = m_{NaOH} = \frac{[NaOH]_{fin} * PM_{NaOH}}{\mathrm{S/L}} = \frac{kg}{kg\ DE} \tag{57}$$

To produce the 2.5M NaOH solution, a certain amount of MilliQ is necessary Equation (58):

$$m_{MilliQ\ to\ add} = m_{NaOH\ 4M} - m_{NaOH} = \frac{kg}{kg\ DE} \tag{58}$$

the EE and CF of both the NaOH solid and the MilliQ used are calculated as follows Equations (59–62):

$$EE_{NaOH} = EErif_{NaOH} * m_{NaOH} = \frac{MJ}{kg\ DE} \tag{59}$$

$$CF_{NaOH} = CFrif_{NaOH} * m_{NaOH} = \frac{kg\ CO_{2eq}}{kg\ DE} \tag{60}$$

$$EE_{MilliQ\ to\ add} = EE_{MilliQ} * m_{MilliQ\ to\ add} = \frac{MJ}{kg\ DE} \tag{61}$$

$$CF_{MilliQ\ to\ add} = CF_{MilliQ} * m_{MilliQ\ to\ add} = \frac{kg\ CO_{2eq}}{kg\ DE} \tag{62}$$

Then it is possible to move on to the calculation of the EE and CF for the treatments performed in the laboratory, that are:

Stirring and heating for t=2h at 80°C (follow the hypothesis 9), Equations (63–64):

$$EE_{Stirrin/Heating} = P_{Stirrin/Heating} * t_{Stirrin/Heating} * Average(EErif_{EtoM}; EErif_{EtoT}) = \frac{MJ}{kg\ DE} \tag{63}$$

$$CF_{Stirrin/Heating} = P_{Stirrin/Heating} * t_{Stirrin/Heating} * Average(CFrif_{EtoM}; CFrif_{EtoT}) = \frac{kg\ CO_{2eq}}{kg\ DE} \tag{64}$$

Centrifugation for t=15 min at environmental temperature (follow the hypothesis 8), Equations (65–66):

$$EE_{Centrifugation} = P_{Centrifugation} * t_{Centrifugation} * EErif_{EtoM} = \frac{MJ}{kg\ DE} \tag{65}$$

$$CF_{Centrifugation} = P_{Centrifugation} * t_{Centrifugation} * CFrif_{EtoM} = \frac{kg\ CO_{2eq}}{kg\ DE} \tag{66}$$

3.2.3. Procedure for the step 2 (acid dropwise)

Table S8. Main data for the step 2 (acid dropwise) for the silica extraction from the raw material.

S/L ratio	$[H_2SO_4]_{fin}$	Density (H_2SO_4 3.5M)	$EErif_{H2SO4\ 98\%}$	$CFrif_{H2SO498\%}$
(kgDE/L)	(mol/L)	(kg/L)	(MJ/kg)	($kgCO_{2eq}$/kg)

Again, the first step is to find the H_2SO_4 mass present inside the solution Equation (67):

$$m_{H_2SO_{4in}} = m_{H_2SO_{4fin}} = m_{H_2SO_4} = \frac{[H_2SO_4]_{fin} * PM_{H_2SO_4}}{\mathrm{S/L}} = \frac{kg}{kg\ DE} \tag{67}$$

To produce the 3.5M H_2SO_4 solution, a certain amount of 98% H_2SO_4 (it is the only one present into the database mentioned at the 1st hypothesis) and MilliQ (ultrapure water) for dilution are necessary Equations (68–69):

$$m_{sol\ H_2SO_4\ 98\%} = \frac{m_{H_2SO_4}}{0.98} = \frac{kg}{kg\ DE} \tag{68}$$

$$m_{MilliQ\ to\ add} = m_{sol\ H_2SO_4\ 5M} - m_{sol\ H_2SO_4\ 98\%} = \frac{kg}{kg\ DE} \tag{69}$$

the EE and CF of both the 98% H_2SO_4 solution and the MilliQ used are calculated as follows, Equations (70–73):

$$EE_{sol\ H_2SO_4\ 98\%} = EErif_{H_2SO_4\ 98\%} * m_{sol\ H_2SO_4\ 98\%} = \frac{MJ}{kg\ DE} \quad (70)$$

$$CF_{sol\ H_2SO_4\ 98\%} = CFrif_{H_2SO_4\ 98\%} * m_{sol\ H_2SO_4\ 98\%} = \frac{kg\ CO_{2eq}}{kg\ DE} \quad (71)$$

$$EE_{MilliQ\ to\ add} = EE_{MilliQ} * m_{MilliQ\ to\ add} = \frac{MJ}{kg\ DE} \quad (72)$$

$$CF_{MilliQ\ to\ add} = CF_{MilliQ} * m_{MilliQ\ to\ add} = \frac{kg\ CO_{2eq}}{kg\ DE} \quad (73)$$

Then it is possible to move on to the calculation of the EE and CF for the treatments performed in the laboratory, that are:

Stirring for t=20 min at environmental temperature (follow the hypothesis 5), Equations (74–75):

$$EE_{Stirring} = P_{Stirring} * t_{Stirring} * EErif_{EtoM} = \frac{MJ}{kg\ DE} \quad (74)$$

$$CF_{Stirring} = P_{Stirring} * t_{Stirring} * CFrif_{EtoM} = \frac{kg\ CO_{2eq}}{kg\ DE} \quad (75)$$

Centrifugation for t=15 min at environmental temperature (follow the hypothesis 8), Equations (76–77):

$$EE_{Centrifugation} = P_{Centrifugation} * t_{Centrifugation} * EErif_{EtoM} = \frac{MJ}{kg\ DE} \quad (76)$$

$$CF_{Centrifugation} = P_{Centrifugation} * t_{Centrifugation} * CFrif_{EtoM} = \frac{kg\ CO_{2eq}}{kg\ DE} \quad (77)$$

Washing (follow the hypothesis 6), Equations (78–79):

$$EE_{MilliQ\_Washing} = EE_{MilliQ} * m_{MilliQ_{Washing}} = \frac{MJ}{kg\ DE} \quad (78)$$

$$CF_{MilliQ\_Washing} = CF_{MilliQ} * m_{MilliQ_{Washing}} = \frac{kg\ CO_{2eq}}{kg\ DE} \quad (79)$$

Drying for t=12h at 80 °C (follow the hypothesis 7), Equations (80–81):

$$EE_{Drying} = P_{Drying} * t_{Drying} * EErif_{EtoT} = \frac{MJ}{kg\ DE} \quad (80)$$

$$CF_{Drying} = P_{Drying} * t_{Drying} * CFrif_{EtoT} = \frac{kg\ CO_{2eq}}{kg\ DE} \quad (81)$$

By adding all the terms of these mechanical / heat treatments and adding the terms of the substances we obtain $EE_{DE} = \frac{MJ}{kg\ DE}$ e $CF_{DE} = \frac{\mathbf{kg}\ CO_{2eq}}{kg\ DE}$ which, having known the % of SiO_2 extracted from Diatomite (about 2-3g of SiO_2), will be transformed into $EE_{SiO_2\_DE} = \frac{MJ}{kg\ SiO_2}$ e $CF_{SiO_2\_DE} = \frac{\mathbf{kg}\ CO_{2eq}}{kg\ SiO_2}$.

3.3. SUB-RAW Index

To obtain the SUB-RAW index, it is necessary to apply the following Equation 82:

$$SUB - RAW\ index = \frac{\left(\log\left(\frac{EE_{SiO_2\_DE}}{MJ/kg}\right) - \log\left(\frac{EE_{SiO_2\_RHPLA}}{MJ/kg}\right) + \log\left(CF_{SiO_2\_DE}\right) - \log\left(CF_{SiO_2\_RHPLA}\right)\right)}{2} \quad (82)$$

4. Phosphorus extraction

4.1. Sub-raw material process

Table S9. Characteristic parameters of the laboratory tools for the phosphorus extraction.

$P_{Stirring}$	$P_{Crushing}$	$P_{Grinding}$	$P_{drying\_halogen}$	Filtration time$_{PR}$	Filtration time$_{RHPLA}$
(W)	(W)	(W)	(W)	(min)	(min)

Table S10. EE and CF for each reference taken in consideration for the phosphorus extraction from the sub-raw material.

EE_{MilliQ}	CF_{MilliQ}	$EE_{TapWater}$	$CF_{TapWater}$	$EErif_{EtoM}$	$CFrif_{EtoM}$	$EErif_{EtoT}$	$CFrif_{EtoT}$
(MJ/kg)	(kg CO_{2eq}/kg)	(MJ/kg)	(kg CO_{2eq}/kg)	(MJ/kg *W*min)	(kg CO_{2eq} /kg *W*min)	(MJ/kg *W*min)	(kg CO_{2eq} /kg *W*min)

4.1.1. Procedure for the step 1

Table S11. Main data for the step 1 (test n. 4) for the phosphorus extraction from the sub-raw material.

S/L ratio	$[HCl]_{fin}$	RHPLA mass	Density (HCl 0.55M)	$EErif_{HCl\,37\%}$	$CFrif_{HCl\,37\%}$
(kgRHPLA/L)	(mol/L)	(g)	(kg/L)	(MJ/kg)	(kgCO_{2eq}/kg)

The first step is to find the HCl mass present inside the solution Equation (83):

$$m_{HCl_{in}} = m_{HCl_{fin}} = m_{HCl} = \frac{[HCl]_{fin} * PM_{HCl}}{\mathrm{S/L}} = \frac{kg}{kg\ RHPLA} \quad (83)$$

To produce the 0.55M HCl solution, a certain amount of 37% HCl (it is the only one present into the database mentioned at the 1st hypothesis) and MilliQ (ultrapure water) for dilution are necessary, Equations (84–85):

$$m_{sol\ HCl\ 37\%} = \frac{m_{HCl}}{0.37} = \frac{kg}{kg\ RHPLA} \quad (84)$$

$$m_{MilliQ\ to\ add} = m_{sol\ HCl\ 1M} - m_{sol\ HCl\ 37\%} = \frac{kg}{kg\ RHPLA} \quad (85)$$

the EE and CF of both the 37% HCl solution and the MilliQ used are calculated as follows Equations (86–89):

$$EE_{HCl\ 37\%} = EErif_{HCl\ 37\%} * m_{sol\ HCl\ 37\%} = \frac{MJ}{kg\ RHPLA} \quad (86)$$

$$CF_{HCl\ 37\%} = CFrif_{HCl\ 37\%} * m_{sol\ HCl\ 37\%} = \frac{kg\ CO_{2eq}}{kg\ RHPLA} \quad (87)$$

$$EE_{MilliQ\ to\ add} = EE_{MilliQ} * m_{MilliQ\ to\ add} = \frac{MJ}{kg\ RHPLA} \quad (88)$$

$$CF_{MilliQ\ to\ add} = CF_{MilliQ} * m_{MilliQ\ to\ add} = \frac{kg\ CO_{2eq}}{kg\ RHPLA} \quad (89)$$

Then it is possible to move on to the calculation of the EE and CF for the treatments performed in the laboratory, that are:

Stirring for t=2h at environmental temperature, Equations (90–91):

$$EE_{Stirring} = P_{Stirring} * t_{Stirring} * EErif_{EtoM} = \frac{MJ}{kg\ RHPLA} \quad (90)$$

$$CF_{Stirring} = P_{Stirring} * t_{Stirring} * CFrif_{EtoM} = \frac{kg\ CO_{2eq}}{kg\ RHPLA} \quad (91)$$

Filtration (follow the hypothesis 3), Equations (92–93):

$$EE_{Filtration} = EE_{TapWater} * m_{TapWater} = \frac{MJ}{kg\ RHPLA} \quad (92)$$

$$CF_{Filtration} = CF_{TapWater} * m_{TapWater} = \frac{kg\ CO_{2eq}}{kg\ RHPLA} \quad (93)$$

Where the $m_{TapWater}$ is the mass of tap water used by the filtration normalized on the RHPLA mass.

By adding all the terms of these mechanical / heat treatments and adding the terms of the substances we obtain $EE_{RHPLA} = \frac{MJ}{kg\ RHPLA}$ e $CF_{RHPLA} = \frac{kg\ CO_{2eq}}{kg\ RHPLA}$ which, having known the % of P extracted from RHPLA (about 3.49% respect the starting material), will be transformed into $EE_{P\_RHPLA} = \frac{MJ}{kg\ P}$ e $CF_{P\_RHPLA} = \frac{kg\ CO_{2eq}}{kg\ P}$

4.2. Raw material process

4.2.1. Procedure for the step 1

Table S12. Main data for the step 1 for the phosphorus extraction from the raw material.

L/S ratio	%HCl in sol.	PR mass	% P_2O_5	Density (HCl 28%)	$EErif_{HCl\ 37\%}$	$CFrif_{HCl\ 37\%}$
(L/kgPR)	(%)	(kg)	(%)	(kg/L)	(MJ/kg)	(kg CO_{2eq}/kg)

The first step is to find the HCl mass present inside the solution Equations (94):

$$m_{HCl_{in}} = m_{HCl_{fin}} = m_{HCl} = \frac{d_{sol\ HCl\ 28\%} * 0.28}{S/L} = \frac{kg}{kg\ PR} \quad (94)$$

To produce the 28% HCl solution, a certain amount of 37% HCl (it is the only one present into the database mentioned at the 1st hypothesis) and MilliQ (ultrapure water) for dilution are necessary Equations (95–96):

$$m_{sol\ HCl\ 37\%} = \frac{m_{HCl}}{0.37} = \frac{kg}{kg\ PR} \quad (95)$$

$$m_{MilliQ\ to\ add} = m_{sol\ HCl\ 28\%} - m_{sol\ HCl\ 37\%} = \frac{kg}{kg\ PR} \quad (96)$$

the EE and CF of both the 37% HCl solution and the MilliQ used are calculated as follows Equations (97–100):

$$EE_{HCl\ 37\%} = EErif_{HCl\ 37\%} * m_{sol\ HCl\ 37\%} = \frac{MJ}{kg\ PR} \quad (97)$$

$$CF_{HCl\ 37\%} = CFrif_{HCl\ 37\%} * m_{sol\ HCl\ 37\%} = \frac{kg\ CO_{2eq}}{kg\ PR} \quad (98)$$

$$EE_{MilliQ\ to\ add} = EE_{MilliQ} * m_{MilliQ\ to\ add} = \frac{MJ}{kg\ PR} \quad (99)$$

$$CF_{MilliQ\ to\ add} = CF_{MilliQ} * m_{MilliQ\ to\ add} = \frac{kg\ CO_{2eq}}{kg\ PR} \quad (100)$$

In addition, the EE and CF of the PR must also be considered, i.e., the mining process until it is brought to the laboratory counter. From the database it is known that $EE_{P_2O_5} = \frac{MJ}{kg\ P_2O_5}$ and $CF_{P_2O_5} = \frac{kg\ CO_{2eq}}{kg\ P_2O_5}$, Equations (101–102):

$$EE_{PR} = \%P_2O_5 * EE_{P_2O_5} = \frac{MJ}{kg\ PR} \quad (101)$$

$$CF_{PR} = \%P_2O_5 * CF_{P_2O_5} = \frac{kg\ CO_{2eq}}{kg\ PR} \quad (102)$$

Then it is possible to move on to the calculation of the EE and CF for the treatments performed in the laboratory, that are:

Crushing for t=10 min:, Equations (103–104):

$$EE_{Crushing} = P_{Crushing} * t_{Crushing} * EErif_{EtoM} = \frac{MJ}{kg\ PR} \quad (103)$$

$$CF_{Crushing} = P_{Crushing} * t_{Crushing} * CFrif_{EtoM} = \frac{kg\ CO_{2eq}}{kg\ PR} \quad (104)$$

Grinding for t=10 min, Equations (105–106):

$$EE_{Grinding} = P_{Grinding} * t_{Grinding} * EErif_{EtoM} = \frac{MJ}{kg\ PR} \quad (105)$$

$$CF_{Grinding} = P_{Grinding} * t_{Grinding} * CFrif_{EtoM} = \frac{kg\ CO_{2eq}}{kg\ PR} \quad (106)$$

Stirring for t=1h at environmental temperature, Equations (107–108):

$$EE_{Stirring} = P_{Stirring} * t_{Stirring} * EErif_{EtoM} = \frac{MJ}{kg\ PR} \quad (107)$$

$$CF_{Stirring} = P_{Stirring} * t_{Stirring} * CFrif_{EtoM} = \frac{kg\ CO_{2eq}}{kg\ PR} \quad (108)$$

Filtration (follow the hypothesis 3), Equations (109–110):

$$EE_{Filtration} = EE_{TapWater} * m_{TapWater} = \frac{MJ}{kg\ PR} \quad (109)$$

$$CF_{Filtration} = CF_{TapWater} * m_{TapWater} = \frac{kg\ CO_{2eq}}{kg\ PR} \quad (110)$$

Where the $m_{TapWater}$ is the mass of tap water used by the filtration normalized on the RHPLA mass.

By adding all the terms of these mechanical / heat treatments and adding the terms of the substances we obtain $EE_{PR} = \frac{MJ}{kg\ PR}$ e $CF_{PR} = \frac{kg\ CO_{2eq}}{kg\ PR}$ which, having known the % of P extracted from PR (about 13.62% respect the starting material), will be transformed into $EE_{P\_PR} = \frac{MJ}{kg\ P}$ e $CF_{P\_PR} = \frac{kg\ CO_{2eq}}{kg\ P}$.

4.3. *SUB-RAW Index*

To obtain the SUB-RAW index, it is necessary to apply the following Equation (111):

$$SUB-RAW\ index = \frac{\left(\log\left(\frac{EE_{P\_PR}}{MJ/kg}\right) - \log\left(\frac{EE_{P\_RHPLA}}{MJ/kg}\right) + \log\left(CF_{P\_PR}\right) - \log\left(CF_{P\_RHPLA}\right)\right)}{2} \quad (111)$$

Table S13. shows the SUB-RAW indices calculated for all 17 tests.

Table S13. Values of the 17 SUB-RAW indeces calculated for the phosphorus extraction from the sub-raw material.

Run Order	Conc HCl (mol/L)	L/S Ratio (mL/g)	SUB-RAW index (/)
1	0.55	30	-0.76
2	0.55	10	-0.43
3	0.1	10	-2.04
4	0.55	50	-0.70
5	0.1	50	-0.78
6	0.1	50	-0.76
7	1	30	-0.84
8	0.1	30	-1.87
9	1	10	-0.34
10	1	50	-0.96
11	1	50	-0.98
12	0.1	10	-1.97
13	0.55	30	-0.74
14	0.55	30	-0.75
15	0.55	30	-0.72
16	1	10	-0.37
17	0.55	30	-0.82
